# Platelet-rich plasma, plasma rich in growth factors and simvastatin in the regeneration and repair of alveolar bone

**DOI:** 10.3892/etm.2013.1327

**Published:** 2013-10-07

**Authors:** CÉSAR RIVERA, FRANCISCO MONSALVE, JUAN SALAS, ANDREA MORÁN, IVÁN SUAZO

**Affiliations:** 1 Unit of Histology and Embryology, Faculty of Health Sciences, University of Talca, Talca 3460000, Chile; 2 Biomedical Sciences Master Program, Oral Pathology, University of Talca, Talca 3460000, Chile; 3 Unit of Physiology and Pharmacology, Department of Basic Biomedical Sciences, Faculty of Health Sciences, University of Talca, Talca 3460000, Chile; 4 School of Dentistry, Faculty of Health Sciences, University of Talca, Talca 3460000, Chile; 5 Autonomous University of Chile, Santiago 7500000, Chile

**Keywords:** platelet-rich plasma, plasma rich-in-growth-factors, simvastatin, MK-733

## Abstract

Platelet preparations promote bone regeneration by inducing cell migration, proliferation and differentiation in the area of the injury, which are essential processes for regeneration. In addition, several studies have indicated that simvastatin (SIMV), widely used for the treatment of hypercholesterolemia, stimulates osteogenesis. The objective of this study was to evaluate the effects of treatment with either platelet-rich plasma (PRP) or plasma rich in growth factors (PRGF) in combination with SIMV in the regeneration and repair of alveolar bone. The jaws of Sprague Dawley rats (n=18) were subjected to rotary instrument-induced bone damage (BD). Animals were divided into six groups: BD/H_2_O (n=3), distilled water without the drug and alveolar bone damage; BD/H_2_O/PRP (n=3), BD and PRP; BD/H_2_O/PRGF (n=3), BD and PRGF; BD/SIMV (n=3), BD and water with SIMV; BD/SIMV/PRP (n=3), BD, PRP and SIMV; and BD/SIMV/PRGF (n=3), BD, PRGF and SIMV. Conventional histological analysis (hematoxylin and eosin staining) revealed that the BD/SIMV group showed indicators for mature bone tissue, while the BD/SIMV/PRP and BD/SIMV/PRGF groups showed the coexistence of indicators for mature and immature bone tissue, with no statistical differences between the platelet preparations. Simvastatin did not improve the effect of platelet-rich plasma and plasma rich in growth factors. It was not possible to determine which platelet preparation produced superior effects.

## Introduction

The repair and regeneration of lost or damaged alveolar bone presents significant problems in dentistry. Previous studies have indicated that the chances of success are increased when the induction of regeneration is induced with autologous compounds, since the use of autologous compounds is associated with an improved prognosis and the absence of the biocompatibility problems that may occur with synthetic graft materials or grafts of a heterologous nature (1,2).

Two platelet preparation products, platelet-rich plasma (PRP) and plasma rich in growth factors (PRGF), have shown positive effects in the regeneration process, as observed in experimental studies (2–6). PRP has demonstrated an ability to improve the regenerative processes (3), accelerate the regeneration of periodontal defects and promote bone formation (4). Moreover, treatment with PRGF following third molar exodontia has been shown to be successful in stimulating bone regeneration. PRGF has also been shown to increase the speed of wound epithelialization of the oral mucosa and improve post-operative recovery (2,5,6).

PRP and PRGF act on already differentiated cells, such as preosteoblasts and osteoblasts; however, they do not exert any effects on the stem cells present in bone tissue, whose differentiation is regulated by bone morphogenetic proteins (BMPs).

It has been shown that drugs of the statins group promote BMP expression, particularly BMP-2, acting indirectly in the bone regeneration process (7,8). One of these drugs, simvastatin (SIMV, also known as MK-733), has been demonstrated to exhibit favorable effects in the treatment of bone remodeling disorders and bone fractures through the promotion of osteogenesis and the reduction of bone resorption (8,9).

Despite the numerous studies describing the benefits of PRP, PRGF and SIMV separately, there has been a lack of investigation into the simultaneous use of these agents. Therefore the main objective of this study was to evaluate the effect of a combined application on the repair of alveolar bone damaged by thermal injury (caused by a rotary instrument) in an animal model.

## Materials and methods

### Study design

This study used an experimental design of randomized blocks. The independent variable was the induction of alveolar bone damage, while the dependent variable was tissue regeneration. All procedures used in the study were in accordance with the guidelines in the Guide for the Care and Use of Laboratory Animals (10) and were approved by the Bioethics Committee of the University of Talca (Talca, Chile).

### Sample size

In order to determine the sample sizes required, a recursive equation was used, as follows: E = N − T − B (10), where E is the degrees of freedom of the error, N is the total degrees of freedom, T is the degrees of freedom of the treatment (number of treatments minus 1) and B is the degrees of freedom between the blocks (block number minus one) (11).

### Experimental groups

Eighteen male, non-consanguineous Sprague Dawley rats, aged 12 weeks and weighing 330–430 g, were obtained with the corresponding health certification from the Institute of Biomedical Sciences (ICBM), University of Chile (Santiago, Chile). The rats were housed at a controlled temperature (22±1°C) under a12-h light/dark cycle (the light was turned on at 8.00 a.m.), with freely available food and water. Each polycarbonate enclosure (20×19×31 cm^3^) contained three rats and was enriched with tissue paper and cardboard rolls (12). The rats were housed at the Animal Facility of the University of Talca.

**Figure 1:**
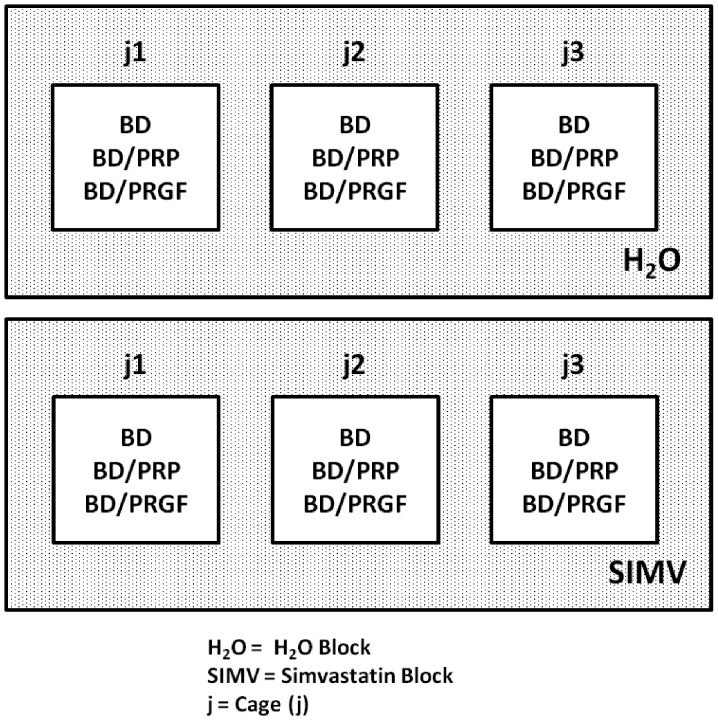
Randomized block design showing random distribution of animals and treatments in each of the cages. H_2_O block, distilled water without the drug; SIMV block, water with SIMV; BD, alveolar bone damage induced by thermal injury; PRP, platelet-rich plasma; PRGF, plasma rich in growth factors.

The rats were divided into six groups, in two blocks (Fig. 1): the H_2_O block (distilled water without the drug) and the SIMV block (water with SIMV). The H_2_O block was subdivided into three groups, as follows: BD/H_2_O (n=3), only alveolar bone damage; BD/H_2_O/PRP (n=3), alveolar bone damage and PRP; and BD/H_2_O/PRGF (n=3), alveolar bone damage and PRGF. The SIMV block was also subdivided into three groups: BD/SIMV (n=3), alveolar bone damage and SIMV; BD/SIMV/PRP (n=3), alveolar bone damage, PRP and SIMV; and BD/SIMV/PRGF (n=3), alveolar bone damage, PRGF and SIMV.

### Procedures

#### Alveolar bone damage induced by thermal injury (BD)

**Figure 2:**
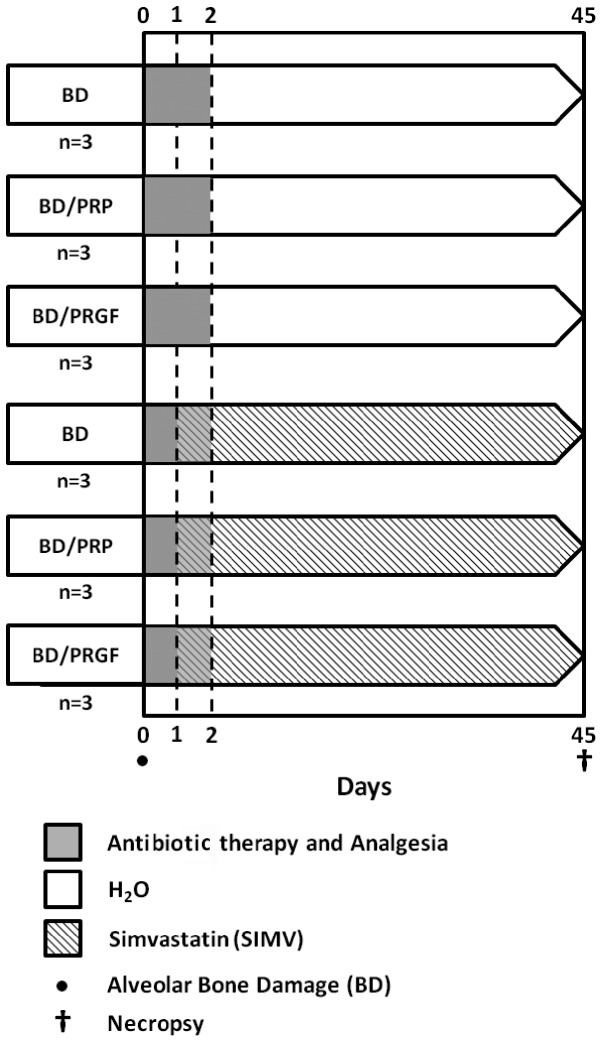
Relevant aspects of the experimental phase, showing group distribution according to the treatment received. PRP, platelet-rich plasma; PRGF, plasma rich in growth factors.

**Figure 3:**
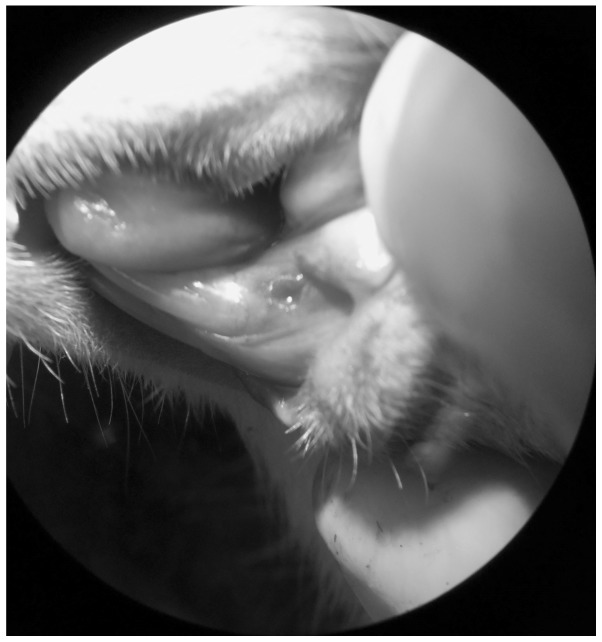
Damage to alveolar bone caused by thermal injury (BD).

The principal procedures of the experimental phase (total, 45 days) are shown in Fig. 2. Prior to the intraoral procedures, the rats were anesthetized with 10% ketamine/2% xylazine/1% acepromazine (Drag Pharma Chile Invetec S.A., Santiago, Chile) at a ratio of 50/5/1 mg/kg, intramuscularly (IM). Having established anesthesia, a thermal injury was induced with a low-velocity 0.8-mm carbide drill at 30,000 rpm, which was oriented in an axial direction, and with constant physiological serum irrigation 2 mm distal to the left lower incisor (Fig. 3). The damage depth was determined with the active tip of the carbide drill.

#### PRP

PRP was obtained by the methods previously described (13), with certain modifications. Briefly, one Sprague Dawley rat was sacrificed in order to obtain sufficient PRP for three surgical procedures. Using cardiac puncture and a full bleed, 1.5 ml blood was obtained, which was collected in a sterile syringe. The blood was deposited into Eppendorf tubes with sodium citrate as an anticoagulant. The tubes were then subjected to an initial centrifugation at 2,900 × g for 10 min. The platelet-rich fraction was separated and centrifuged again at 600 × g for 15 min. For platelet activation, the plasma was mixed for 60–90 sec using bovine thrombin (Thermo Scientific^®^, Thermo Fisher Scientific, Inc., Waltham, MA, USA) to produce a mixture with a gelatinous consistency that was applied to the mandibular bone defects. The edges of the incision were brought together with sutures, in order to prevent the displacement of the gel.

#### PRGF

PRGF was obtained by the methods described previously (14), with modifications. One Sprague Dawley rat was sacrificed in order to obtain sufficient PRGF for ten surgical procedures. Using cardiac puncture, 1.5 ml blood was collected in a sterile syringe (13). The blood was then placed into Eppendorf tubes with 3.8% sodium citrate solution as an anticoagulant, prior to the tubes being exposed to centrifugation at 300 × g for 8 min. With this process, the blood components were separated into three phases. Phase I, the most superficial, corresponded to the phase of platelet-poor plasma. This was followed by Phase II, which had an intermediate concentration of platelets, similar to that observed in circulating blood, and then Phase III, which was located immediately above the white phase of leukocytes and was the phase with the greatest concentration of growth factors. Using a sterile pipette, the first two phases were eliminated and the growth factor-rich Phase III was then transferred to a new Eppendorf tube for activation with 10% calcium chloride (ratio of 0.05 ml CaCl per 1 ml plasma). From this procedure, a substance with a gelatinous consistency was produced, which was applied to the bone defects of the left mandibular arch. The edges of the incision were brought together with sutures to prevent the displacement of the gel.

#### Post-surgical pharmacological therapy

Following surgery, the animals received antibiotic therapy, comprising 16,000 UI benzathine benzylpenicillin, IM, and anti-inflammatory therapy, comprising 500 mg/500 ml acetaminophen, which was administered via the common water during the first two days subsequent to surgery, in accordance with the previously used protocol (15).

#### SIMV

Following previously published guidelines and methods (16,17), SIMV (Simvass; Laboratorios Rider S.A., Santiago, Chile) was orally administered as a SIMV suspension diluted in distilled water, at a dose of 20 mg/kg/day, for 6 weeks. Free administration, using opaque bottles, commenced the day subsequent to the induction of the bone damage by thermal injury.

#### Sample collection

At the end of the experimental phase, the animals were sacrificed by an overdose of anesthetics. The jaws of the rats were hemisectioned and then fixed with 10% formalin, prior to dentoalveolar segments, containing alveolar bone, the left lower incisor and gingival tissue, being obtained. Tissue blocks from the left sectioned jaw were dehydrated with 5% nitric acid for seven days, in order to perform a subsequent conventional histopathological analysis using hematoxylin and eosin (H&E) staining.

#### Histological evaluation

The histological analysis was conducted by an academic (CR) from the University of Talca. The observer was unaware of the group to which the study samples belonged (single-blind model). For the histological analysis of the preparations, a Carl Zeiss Primo Star Trinocular (stereo) microscope (Carl Zeiss MicroImaging Inc., Gottingen, Germany) was used. Images were captured using a digital camera connected to the microscope and then connected to a computer. A qualitative analysis was performed for the histological assessment of the samples, in order to determine the presence or absence (dichotomous variable) of nine parameters for the regeneration/repair of bone, based on the texts by Leeson *et al*(18) and Gartner and Hiatt (19) (Table I).

### Statistical analysis

A χ^2^ test was used for the statistical analysis, with certain corrections according to the expected frequencies in the contingency tables. P<0.05 was considered to indicate a statistically significant difference.

## Results

**Figure 4:**
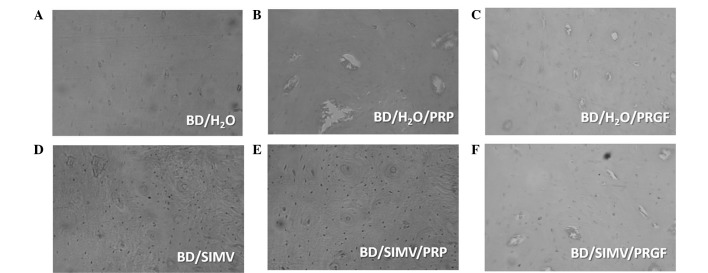
Representative microphotographs of the study groups. BD, alveolar bone damage induced by thermal injury; PRP, platelet-rich plasma; PRGF, plasma rich in growth factors; SIMV, simvastatin.

Following the histological procedure, 36 plaques were selected for analysis (six representatives for each study group). The histological characteristics for each group are shown in Fig. 4.

### General histological characteristics

In group BD/H_2_O there was a dominance of mature osteons with a well-defined structural order, characterized by high mineralization and an absence of immature structures. Group BD/PRP showed the presence of osteons in different phases of maturation, in addition to globular (large) and ellipsoidal osteocytes. In group BD/PRGF an equal presence of mature and immature osteons was observed, in addition to large globular and ellipsoidal osteocytes. Group BD/SIMV exhibited the presence of mature osteons with a narrow central channel, ellipsoidal osteocytes and a high inorganic component. Group BD/SIMV/PRP showed the presence of osteons at different stages of maturation with narrow central channels and ellipsoidal osteocytes. In group BD/SIMV/PRGF, immature osteons with a central channel and blood vessels were observed, in addition to ellipsoidal osteocytes.

### Relevant comparisons

Table II shows the features observed in the histological analysis. Mature osteons were observed in the plates of all the groups; however, statistically significant differences were apparent between groups BD/H_2_O/PRP and BD/SIMV/PRP (67% and 100% presence, respectively; χ^2^ test; P=0.035). In groups BD/H_2_O/PRP, BD/H_2_O/PRGF and BD/SIMV/PRGF there was a 100% presence of immature osteons, while the BD/H_2_O and BD/SIMV/PRP groups were significantly different with 17 and 33% presence, respectively (χ^2^ test; P≤0.05). In group BD/SIMV, no immature osteons were observed. In group BD/H_2_O/PRP there was a 50% presence of random collagen fibers, while in group BD/H_2_O random collagen fibers were absent from all the plates examined. The latter result was precisely the opposite of that observed for ordered collagen fibers, which were present in all the examined plates (χ^2^ test; P=0.046). The BD/H_2_O/PRP group showed a 100% presence of large, globular osteocytes, which was significantly different to the BD/H_2_O/PRGF group, in which the presence was 50%, and these osteocytes were absent from the BD/H_2_O group (χ^2^ test; P≤0.05). Group BD/SIMV/PRP exhibited globular osteocytes in 67% of the plates, which was statistically different from group BD/SIMV, in which no globular osteocytes were present (χ^2^ test; P=0.038). No statistically significant differences where observed between the groups that used platelet preparations (BD/H_2_O/PRP and BD/H_2_O/PRGF) and those treated with platelet preparations plus SIMV (BD/SIMV/PRP and BD/SIMV/PRGF). Groups BD/H_2_O/PRGF and BD/H_2_O/PRP exhibited a 50% presence of osteoid tissue, while no osteoid tissue was observed in group BD/H_2_O (χ^2^ test; P=0.046). With regard to the characteristic of a highly inorganic matrix, the BD/H_2_O group showed a 100% presence, while in group BD/H_2_O/PRGF the presence had decreased to 50% (χ^2^ test; P=0.046).

## Discussion

For the histological assessment, nine variables were analyzed, corresponding with the morphological characteristics of normal bone tissue (19–21) and the indicators for bone regeneration. The groups treated with BD and PRP (BD/H_2_O/PRP) and with BD and PRGF (BD/H_2_O/PRGF) showed the presence of osteons with central (Haversian) channels, random collagen fibers, large, globular osteocytes and a lower degree of mineralization compared with the control group (BD/H_2_O). The presence of these variables, primarily the presence of osteons, whether mature or immature, was indicative of bone tissue, since osteons are the basic structural unit of this type of tissue (or BSU, Bone Structural Unit, according to Geneser, 2008).

Defining the concept of repair as the restoration of a lesion by a tissue that differs from the original in morphology and function, with the existence of a greater presence of fibrous tissue (22), it was proposed that the BD/H_2_O/PRP and BD/H_2_O/PRGF groups exhibited a healed bone tissue that was a result of regeneration and not reparation. This was due to the fact that, in all the groups, the new-formed tissue was indistinguishable from the original tissue (23).

The results of this study were consistent with those of previous studies (4,13,24), in which it was concluded that platelet preparations comparable with those used in the current study favor and accelerate bone regeneration. When comparing the BD/PRR and BD/PRGF groups, the results showed no statistically significant differences in histological variables, and the features observed were similar in the two groups. This suggested that there was not likely be any difference between the use of either platelet preparation (according to the analysis in the present study) and that regeneration indicators were likely to remain essentially the same, irrespective of the preparation used to treat the damage. Furthermore, it was indicated that PRP or PRGF were likely to result in regeneration based on a possible bone adaptable for masticatory function.

In this study, a second block of groups was treated with 10 mg SIMV, orally, for a period of 42 days. The results suggested that SIMV exerted a favorable effect on bone regeneration, since the histological parameters analyzed revealed the formation of a mature compact bone, with the presence of organized, mature osteons with a high mineral content.

SIMV, while acting on the mevalonate pathway, generates increased bone formation and decreased bone resorption (9,25). This may explain the results of the histological analysis for the samples of the BD/SIMV group, in which the bone tissue was observed to be mature, highly organized (100% presence of mature osteons), with the presence of central channels (100% presence), organized osteocytic lacunae (100% presence of ellipsoidal osteocytes), a high mineral content (100% presence of inorganic matrix) and ordered collagen fibers (83% presence). These results were consistent with those of a previous study, in which it was concluded that SIMV had the potential to increase and maintain osteoblastic function, allowing the regeneration of lost alveolar bone in rats with induced periodontitis (26).

Another study evaluated the efficacy of the oral administration of SIMV in the process of repairing defects in the tibia of ovariectomized rats (17). SIMV had a favorable effect on bone regeneration; this was observed in a group of rats treated with the drug, where the bone formation was higher than that in the control group, similar to other prior results (27). The results were indicative of high osteoblastic activity and osteoclast scarcity, which showed the action of the SIMV. By inhibiting the 3-hydroxy-3-methylglutaryl-coenzyme A (HMG-CoA) reductase enzyme involved in the mevalonate pathway, SIMV stimulates osteoblastic differentiation and promotes the expression of BMP-2, while simultaneously decreasing the process of bone resorption by blocking the prenylation of certain signaling molecules that are essential for osteoclastic activity (28,29). This was consistent with the favorable effect of SIMV on bone tissue regeneration observed in the BD/SIMV group, since the tissue exhibited features of mature or secondary-type bone tissue and was highly organized with a significant mineral content.

A number of studies have investigated the effect of SIMV on bone regeneration, experimentally and clinically, and have revealed positive results (9,30,31), while others have observed no beneficial effects (32–34). These contradictory results may be associated with the different doses used. A previous study suggested that the dose pattern affects bone formation and resorption (16). This study, by Maritz *et al*, measured the effect of different doses of orally administered SIMV (1, 5, 10 and 20 mg/kg/day) in ovariectomized rats, and demonstrated that high doses of the drug (20 mg/kg/day) equitably increased bone formation and resorption, whereas a low dose (1 mg/kg/day) decreased formation and increased resorption (16). Therefore, the effects of SIMV on bone tissue appeared to be directly dependent on the employed dose, which may explain the contradictory results from other studies. A further explanation may be that the intensity of the stimuli (pressure and heat of carbide drill) used was different.

When comparing the results obtained in the present study for the BD/SIMV and BD/SIMV/PRP groups, it was observed that the combination of PRP and SIMV produced better indicators for bone regeneration than SIMV alone, due to the coexistence of characteristics of mature bone tissue and a bone remodeling phase.

In group BD/SIMV/PRP, increases in the majority of the histological evaluation parameters for bone regeneration were observed. Consistent with the observations for the BD/SIMV group, mature osteons, ellipsoidal osteocytes, organized collagen fibers and inorganic matrix were present, which indicated that the regenerated bone tissue in group BD/SIMV/PRP corresponded to a mature or secondary-type bone tissue. Moreover, unlike the BD/SIMV group, there was an increase in the presence of immature osteons, ordered collagen fibers, large, globular osteocytes and central channels (the latter two being statistically significant), which suggested that a remodeling process was occurring in the bone tissue, typical of a functional type of bone subjected to mechanical loads. Therefore, due to the coexistence of the features of mature and immature bone tissue, it was proposed that the regenerated bone tissue in the BD/SIMV/PRP group corresponded to a mature or secondary remodeled bone tissue (20).

Since the combination of these two platelet preparations and SIMV in drinking water has not been studied previously, it is not possible to perform an appropriate comparison with other studies. However, as the therapeutic actions of the platelet preparations have been demonstrated to be based on processes for modulation and the acceleration of scarring through the growth factors present in platelets (23), the results obtained in the BD/SIMV/PRP group may be compared with studies using PRP.

Simman *et al*(13) evaluated the therapeutic role of PRP in the repair of fractures induced in the tibias of Lewis rats. In their study, it was determined that PRP accelerated fracture repair, since four weeks subsequent to the administration of PRP the histological analysis revealed that the group treated with PRP showed greater bone formation. Furthermore, this result was consistent with that of the radiographic analysis of the fractures, which also showed higher callus formation in the PRP-treated group than in the control group. It was observed that the fracture healing in rats was dependent on the local expression of growth factors and BMPs, which act on osteoblast differentiation and promote osteogenesis. However, it has been suggested that growth factors only act to promote the proliferation and differentiation of preosteoblasts and osteoblasts, and are unlikely to act on ‘adult’ stem cells present in the bone tissue, whose differentiation is regulated by BMPs (23). Based on this, the combination of PRP with SIMV has been demonstrated to promote an increase in BMP expression, particularly BMP-2 expression, leading to increased osteoblast activity by inducing the differentiation of stem cells into osteoblast cells (7,8). Consistent with this, the results obtained in the BD/SIMV/PRP group indicated that PRP and SIMV exerted a favorable effect on bone regeneration, based on the greater presence of histological features of regeneration in the BD/SIMV/PRP group than in the BD/SIMV group.

When comparing the histological parameters of bone regeneration in the BD/SIMV and BD/SIMV/PRGF groups, it was demonstrated that the combination of PRGF and SIMV produced the best indicators of bone regeneration, with characteristic areas of mature bone tissue coexisting with immature bone tissue. This suggested a process of bone remodeling.

Consistent with the results for the BD/SIMV/PRP group, in the BD/SIMV/PRGF group there was an increase in the majority of the histological evaluation parameters for bone regeneration. In comparison with the BD/SIMV group, it was observed that there was mature bone tissue, which was organized with a high mineral content, and a presence of mature osteons, ellipsoidal osteocytes and an inorganic matrix. The increased presence of immature osteons and central channels (statistically significant), large, globular osteocytes, ordered collagen fibers and osteoid tissue were indicative of the remodeling process in bone tissue and characteristic of a functional-type bone. These results indicate that the biologically regenerated bone, stimulated by the effects of PRGF and SIMV, was a mature bone tissue or a bone of secondary remodeling, comparable with that observed in the BD/SIMV/PRP group.

A notable previous study used platelet-derived growth factor (PDGF) and SIMV in microspheres, in order to overcome the injury induced by the heat generated during the osteotomy while placing dental implants in rats (35). In the absence of irrigation, a significant reduction of cell viability and an increase in inflammation and bone formation were observed, without evidence of osteogenesis. SIMV and PDGF treatments facilitated cell viability and the reduction of osteonecrosis, while the combination of the two treatments further increased the signs of osteogenesis and bone maturation in a sequential treatment modality, which showed the positive effect of platelet products and this derivative of lovastatin.

In the present study, in which qualitative variables were used to analyze the results and compare the combination of PRP and SIMV with PRGF and SIMV, it was not possible to determine which platelet preparation produced superior effects in the process of bone regeneration, due to the fact that no statistically significant differences were observed between the two treatments.

The essential limitation of this study was the lack of quantitative data. Therefore, future interventions are required, which consider similar variables to the present study, in addition to discriminating the type of new bone and using a histomorphometric analysis and/or cell count. It is further recommended that serial sacrifices be performed, with an increased number of experimental animals, in order to provide sufficient temporal evidence of the regeneration process/alveolar bone repair.

## Figures and Tables

**Table I:** Bone regeneration/reparation parameters.

Parameter	Description	Primary bone tissue	Secondary bone issue	Remodeled secondary bone tissue
Mature osteon	Basic bone unit (UBO) present in secondary bone tissue, with defined order	−	+	+
Immature osteon	Basic bone unit (UBO) present in primary bone tissue, undefined order	+	−	+
Central channels	Central channel observable in the osteon (both mature and immature)	+	+	+
Random collagen fibers	Collagenous fibers directed in all directions, not well-defined sheets	+	−	+
Organized collagen fibers	Collagen fibers of parallel course arranged on concentric lamellae	−	+	+
Globular osteocytes	Young osteocytes easily observable under the microscope, contained in rounded lagoons between poorly defined lamellae	+	−	+
Ellipsoidal osteocytes	Mature osteocytes contained in ellipsoidal lagoons, visible between ordered lamellae	−	+	+
Highly inorganic matrix	Presence of mineralized matrix	−	+	+
Osteoid tissue	Presence of non-mineralized organic matrix	+	−	+

**Table II:** Count of histological features in each treatment.

	Treatments											
	
	BD/H_2_O		BD/H_2_O/PRP		BD/H_2_O/PRGF		BD/SIMV		BD/SIMV/PRP		BD/SIMV/PRGF	
						
Features	P	A	P	A	P	A	P	A	P	A	P	A
Mature osteon	6	0	4	2	6	0	6	0	6	0	6	0
Immature osteon	1	5	6	0	6	0	0	6	2	4	6	0
Central channels	6	0	6	0	6	0	6	0	6	0	6	0
Random collagen fibers	0	6	3	3	3	3	1	5	1	5	0	6
Organized collagen fibers	6	0	5	1	3	3	5	1	6	0	6	0
Globular osteocytes	0	6	6	0	3	3	0	6	4	2	1	5
Ellipsoidal osteocytes	5	1	5	1	2	4	6	0	6	0	5	1
Highly inorganic matrix	6	0	4	2	3	3	6	0	6	0	5	1
Osteoid tissue	0	6	3	3	3	3	0	6	0	6	0	6

BD, alveolar bone damage induced by thermal injury; PRP, platelet-rich plasma; PRGF, plasma rich in growth factors; SIMV, simvastatin; P, present; A, absent.
